# Treatment of contagious bovine pleuropneumonia as a potential driver for antimicrobial resistance in pastoral production systems of Kenya

**DOI:** 10.1038/s41598-026-41713-x

**Published:** 2026-03-04

**Authors:** James M. Akoko, Noah O. Okumu, Angela Makumi, Dishon Muloi, Hussein M. Abkallo, Hezron Wesonga, Winnie Chebore, Juliet Masiga, Gordon Nguka, Elise Schieck, Musa M. Mulongo

**Affiliations:** 1 International Livestock Research Institute, Nairobi, Kenya; 2 University of Nairobi, Nairobi, Kenya; 3 Masinde Muliro University of Science and Technology, Kakamega, Kenya

**Keywords:** Diseases, Health care, Microbiology

## Abstract

The control of contagious bovine pleuropneumonia (CBPP) remains constrained by institutional, financial and technical barriers in many livestock-keeping regions of sub-Saharan Africa, despite its detrimental impact on trade, pastoral livelihoods and food security. Therefore, livestock keepers are relying on suboptimal use of antimicrobials to manage CBPP outbreaks. We conducted an outbreak investigation to understand epidemiological and management practices in 95 herds with ongoing or recent cases of CBPP in five counties of Kenya. A wide range of antimicrobials including tetracycline, penicillin-streptomycin (pen & strep), tylosin, and diminazen were used in majority of herds (90/95; 94.7%), with half of these herds receiving sequential administration of two or more types of drugs to address failure of the first use. Only 33/95 (34.7%) of farmers reported to have vaccinated their animals and most of them vaccinated in response to outbreaks in their herds rather than the expected routine preventive program (28/33, 84.8%). The average case fatality rate (CFR) for CBPP was estimated at 36%, with only treatment with tylosin resulting in a significant reduction in mortalities due to CBPP (*p* = 0.035). Majority of livestock keepers (83.3%) continued to consume milk from their animals during and after the treatment period, without observing the stipulated withdrawal periods. Policy emphasis should shift toward prudent use of antimicrobials and preventive vaccination as the cornerstone of CBPP control, while using multifaceted One Health strategy that bridges veterinary, public health, and environmental sectors.

**Supplementary Information:**

The online version contains supplementary material available at 10.1038/s41598-026-41713-x.

## Introduction

Contagious bovine pleuropneumonia (CBPP) is a highly contagious transboundary respiratory disease of cattle caused by *Mycoplasma mycoides* subsp. *mycoides* (*Mmm*). The disease presents a major constraint to livestock productivity, trade, and food security in sub-Saharan Africa (SSA), particularly within pastoral and agropastoral production systems where livestock are central to livelihoods^1,2^. In endemic areas, morbidity rates average 30%^3^, but can reach up to 100% in naïve herds. CBPP leads to substantial reductions in milk and meat production, resulting in food insecurity, high costs of treatment, and expenses associated with restocking following death or culling, with mortality rates reaching up to 80% during sporadic and poorly tracked outbreaks^4^. The persistence and spread of CBPP are driven by communal grazing, unrestricted animal movement, and seasonal transhumance^3^. In addition to direct production losses, the disease causes significant indirect economic losses through trade restrictions at local, national, and international levels, which in turn depress the cost of livestock and livestock products and negatively affect livelihoods, food and nutrition security.

CBPP eradication in high-income countries (HICs) was successfully achieved through programs such as stamping-out^5^. However, implementing this strategy in the wider parts of SSA remains challenging due to weak animal health service infrastructure, and socio-economic as well as cultural constraints that limit disease reporting and compliance. As a result, vaccination using live attenuated *Mmm* strains such as T1/44 and T1sR has become the principal control strategy in endemic SSA regions^6^.

Despite the central role of vaccination, vaccine performance and uptake are limited by logistical and biological factors, including poor cold-chain infrastructure, inadequate coverage, short-duration of immunity (6 months), and post-vaccinal adverse inflammatory reactions at the site of injection that undermine farmer confidence in vaccines^7,8^. These constraints, coupled with the difficult to implement test and slaughter methods, have partly led to increased reliance on antibiotics as a means of managing outbreaks and reducing severity of clinical disease. Thus antimicrobial treatment of CBPP-infected cattle persists, although it is not recommended by World Organization for Animal Health (WOAH), as it can lead to the formation of chronic carriers that remain infectious and complicate long-term eradication efforts^5^.

In the absence of reliable vaccines and diagnostic support, livestock keepers often administer antimicrobials on their own to manage suspected CBPP cases. This practice has contributed to widespread misuse, manifested as overuse (prophylactic herd-level treatments), underuse (sub-therapeutic dosing or premature cessation of therapy), and inappropriate drug selection^9,10^. Notably, beta-lactam antibiotics such as ampicillin and penicillin are known to be ineffective against *Mycoplasma* species due to the absence of a cell wall (the target of these antibiotics), but are still commonly used owing to their affordability, accessibility, and perceived efficacy^11,12^. Such misuse undermines treatment outcomes, facilitates persistence of infection, and potentially promotes the emergence and spread of antimicrobial resistant (AMR) pathogens, a major public health problem globally.

Beyond animal health, irrational antimicrobial use poses significant public health risks through contamination of animal-source foods^13,14^. In pastoral communities, milk serves as a critical source of nutrition and income, and withdrawal periods following antimicrobial therapy are rarely observed. Consequently, milk contaminated with antimicrobial residues frequently enters the human food chain and contributes to the development of AMR bacteria amongst zoonotic and commensal species^15,16^.

This study investigated the epidemiology and management of CBPP in Kenya’s pastoral settings and reports the patterns and practices associated with antimicrobial use in the management of CBPP within Kenya’s pastoral production systems. The study also highlights continued consumption of milk during and after administration of antimicrobials which poses a potential public health risk.

## Methods

**Fig. 1:**
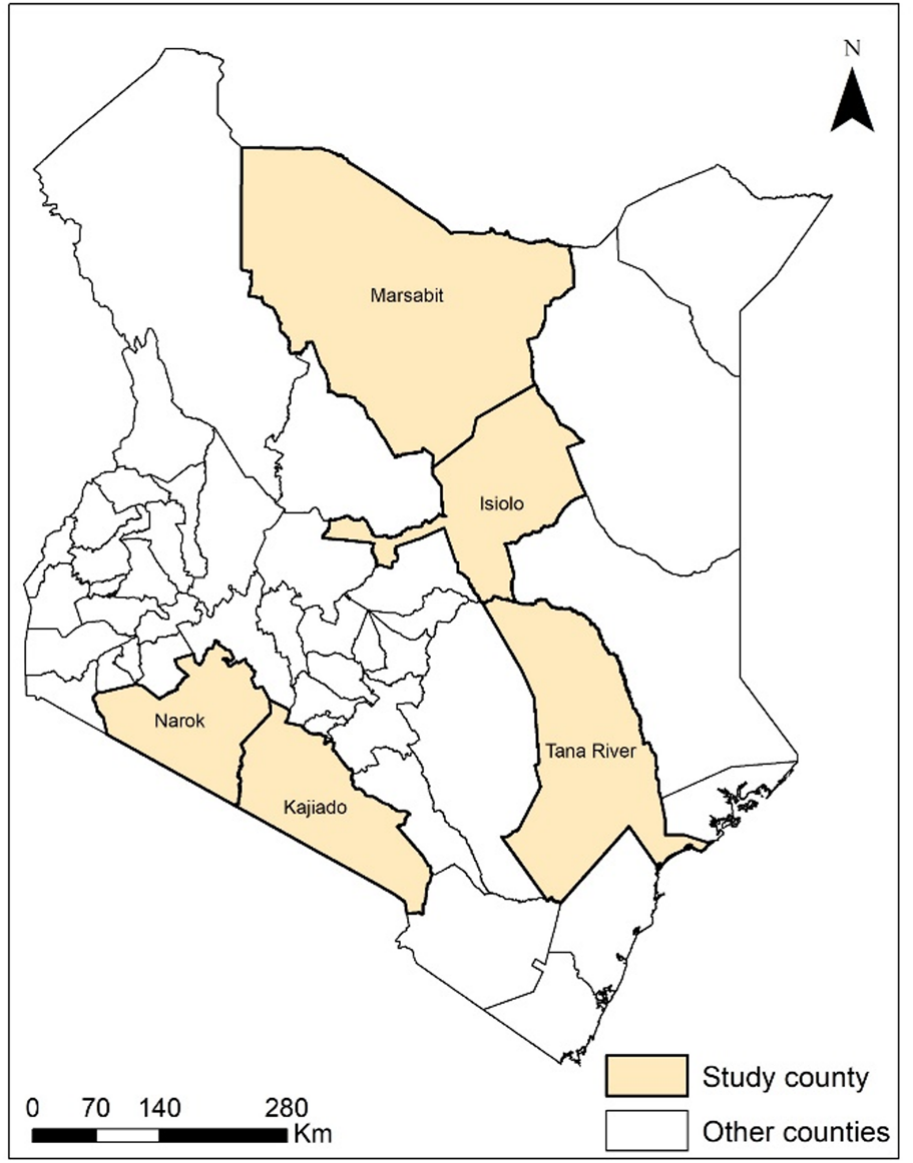
Map of Kenya showing the five study counties. The QGIS version 3.40 was used to generate this map, using the free shapefile downloaded from https://gadm.org/download_country.html.

A cross-sectional study was conducted to investigate the epidemiology and management practices of CBPP in cattle herds in Marsabit, Isiolo, Tana River, Kajiado and Narok counties in Kenya (Fig. 1). The selection of these counties was based on the occurrence of CBPP outbreaks reported and confirmed by county veterinary authorities within the past two years. Livestock keeping serves as the primary source of livelihood, across all five counties, where nomadic pastoralism is the predominant system of production, in these largely remote and arid regions (with a few areas characterized by semi-arid and medium- or high-production conditions).

The study was conducted in July 2025 and included all CBPP cases that occurred from January 2022. Within each county, herds confirmed as CBPP positive through a positive laboratory test result, and those suspected of being CBPP positive upon clinical examination by qualified veterinary officers were used as index cases. These herds were visited for a short interview with the household head to confirm if the clinical history or presentation was consistent with CBPP. Upon confirmation, these index cases were used as entry points for a snowball sampling approach, where herd owners referred the research team to neighboring herds that had experienced similar clinical signs or disease episodes. This approach allowed the inclusion of all the affected herds in each area, and subsequent capturing of the local occurrence patterns and management responses.

Data was collected using a newly developed, pretested semi-structured questionnaire administered through the Open Data Kit (ODK) mobile platform^17^. The questionnaire captured detailed information on herd demographics, disease history, clinical symptoms observed, number of affected animals, management and control measures undertaken and disease outcomes (recoveries and mortalities). In addition, the respondents were asked about antimicrobial use practices, including types of antimicrobials used, sources of antimicrobials, administration frequencies for each drug, perceived effectiveness of treatment, and information on withdrawal of milk consumption following treatment.

### Statistical analysis

Data was cleaned and analyzed in R statistical software (version 4.4.2). Descriptive statistics were computed to summarize management practices, drugs used and their corresponding frequency of administration, and outbreak outcomes. The proportion of diseased animals, as reported by the herd owners, (number of diseased divided by total number of animals in a herd) was analyzed to get the cumulative incidence during the period when livestock keepers observed clinical presentation of CBPP in their herds. Additionally, the Multiple Linear Regression (MLR) was used to model the relationship between the proportion of animals that die after treatment and the various individual drugs, by using proportion of deaths as the outcome indicators and the drugs (tetracycline, penicillin-streptomycin, tylosin, and diminazen) as predictors. Similarly, the proportion of deaths amongst diseased animals was modelled using a Generalized Linear Model with a two-column binomial response (deaths and survivors), using a quasi-binomial family and logit link to generate case fatality rate (CFR).

### Ethics consideration

#### Ethical approval

for this study was obtained from the International Livestock Research Institute, Institutional Ethics Committee (Ref: ILRI-IREC2023-73) and the National Commission for Science, Technology and Innovation in Kenya (NACOSTI/P/24/32686). All methods were performed in accordance with the relevant guidelines and regulations stipulated by the two authorizing institutions. Participation was voluntary, and informed consent was obtained from all respondents. All data was anonymized prior to analysis to ensure confidentiality of participants and their herds.

## Results

### Study area, herd demography, disease burden, and control measures

We conducted this study in five counties and enrolled 95 herds. Out of the 95 herds, 45 had active clinical CBPP cases at the time of data collection, while 50 had history of the disease within the past two years. The average number of cattle in a herd (herd size) was estimated at 53, with the smallest herd having 3, while the largest had 500 cattle. The herd-level cumulative incidence of CBPP was at 39.9%, with each additional animal in a herd corresponding to a small but measurable increase in disease occurrence (*p* = 0.009). In addition, the average duration for the disease to clear in a herd was reported to be 11 months (ranging from 1 to 40 months), and majority of livestock keepers (80/95) either sold cattle, sheep, or goats to generate money for disease control during active disease. No routine vaccination program for CBPP was present in the study population, either at the herd or county level. Adoption of vaccination for CBPP control was low with only 33/95 (34.7%) farmers reporting to have vaccinated their animals, mostly in response to outbreaks in their herds rather than the expected routine preventative program (28/33, 84.8%).

**Fig. 2:**
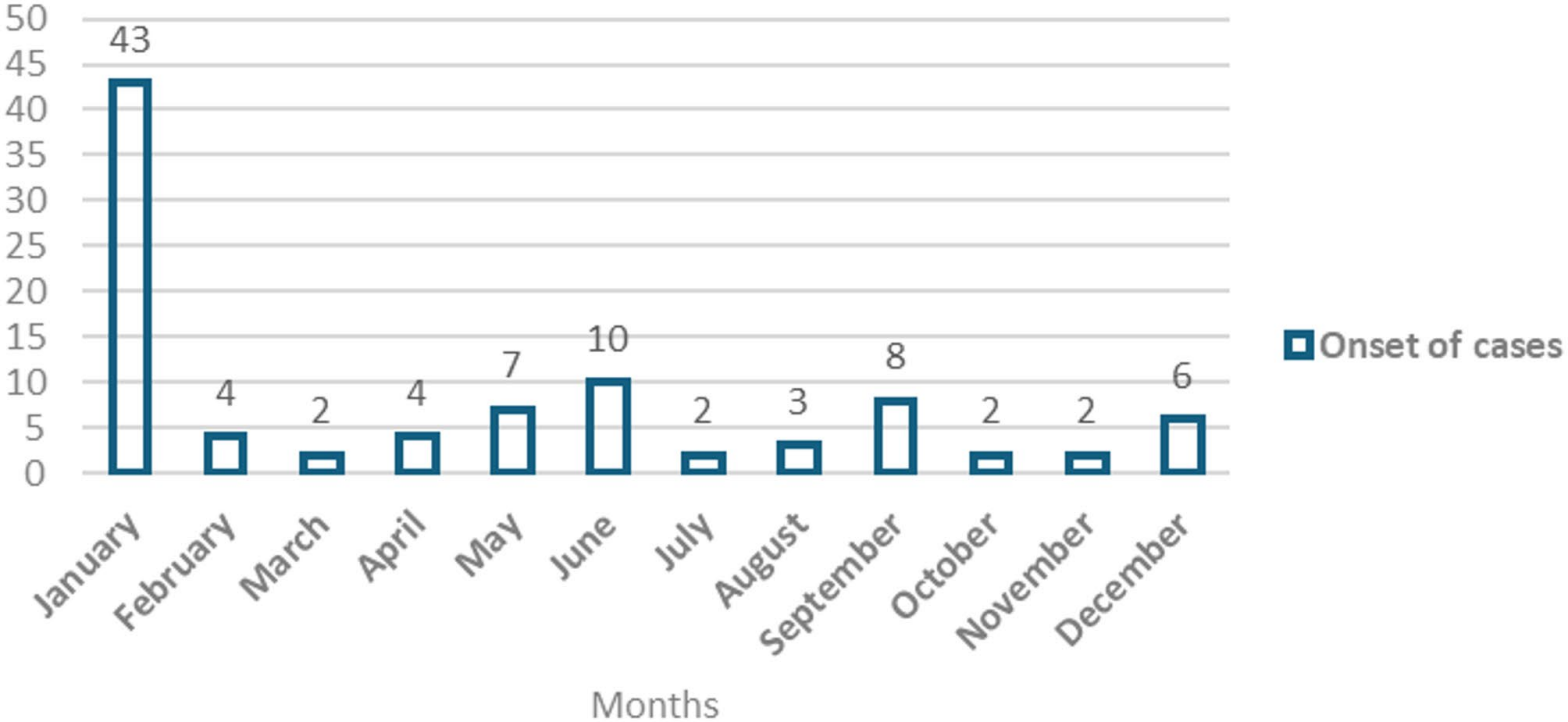
Showing the number of herds with onset of clinical CBPP cases in the different months of the year.

The monthly onset of clinical CBPP cases at the herd level varied across different months of the year, the highest onsets recorded in January at 45.3% (43/95), followed by June 10.5% (10/95), and September 8.4% (8/95), while the rest were spread across other months (Fig. 2).

### Analysis of CBPP treatment in different herds reveals a diverse and sequential use of antimicrobials

**Fig. 3:**
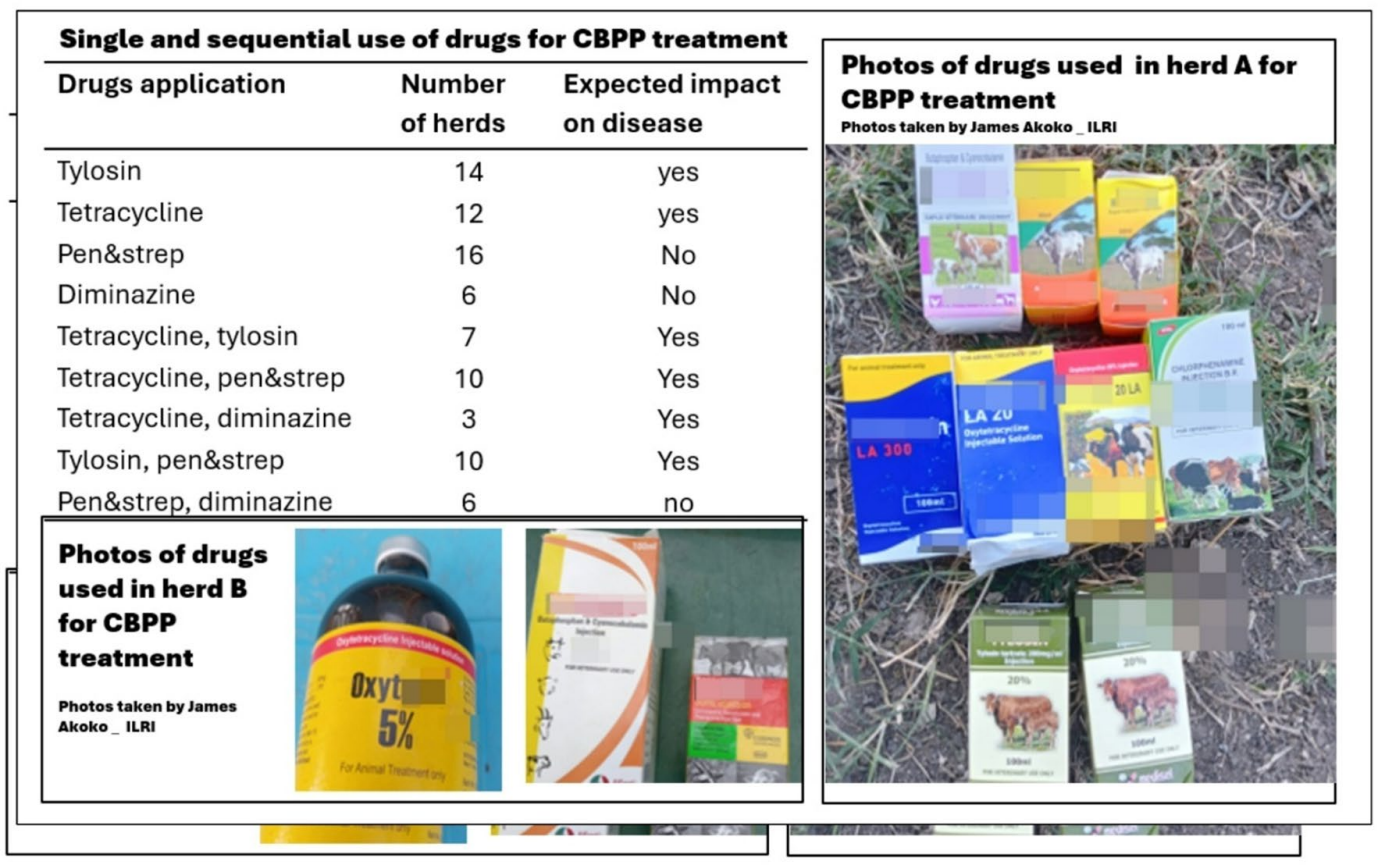
Summary of the different antimicrobial applied in singular or sequentially for treatment of CBPP.

Most livestock keepers (90/95; 94.7%) acknowledged using antimicrobials to treat CBPP. A range of drugs including different concentrations of tetracycline (5%, 10%, 20% and 30%), penicillin-streptomycin (pen & strep), tylosin, and diminazen (an antiprotozoal drug) were used to “treat” *Mmm* infection. Also administered were dewormers, multivitamins, and antihistamine. The list of administered drugs varied between herds, and half of the livestock keepers reported the sequential administration of two or more types of drugs to address failure of the first use (Fig. 3).

NB: Expected impact on disease, is the expected outcome after treatment with a given drug or combination, based on their mode of action and effectiveness against *Mmm*.

### Dosing of antimicrobials and posttreatment milk consumption

Different antimicrobials are commonly administered to control respiratory diseases such as CBPP. However, the administration rates reported by livestock farmers indicated both underuse and overuse when compared with the treatment durations observed among professional practitioners in the study region (Table 1). Moreover, the investigation revealed that majority of livestock keepers (83.3%) continued to consume milk from their animals during and after the treatment period, without observing stipulated withdrawal periods.

**Table 1:** Analysis of drug administration frequencies show the existence of underuse, overuse and consumption of milk from treated animals without observing withdrawal periods.

Drug	Number of treatment days as per the common practice by area veterinarians	Number of treatment days (No of herds)	% non-withdrawal of milk consumption after treatment
Oxytetracycline Long Acting	1–2	1 (1)	82.9% (58/70)
		3 (15)	
		4–7 (10) ^***^	
Oxytetracycline Short acting	3	1–2 (7) *	
		3 (1)	
		4–7 (7) ***	
Tylosin	3	1–2 (14) *	92.% (4/55)
		3 (23)	
		4–7 (14) ***	
Pen & strep	Not used	1–2 (14)	88.6% (31/35)
		3 (17)	
		4–7 (10)	
Diminazen	Not used	1–2 (13)	83.3% (15/18)
		3 (4)	
		4 (1)	

* Indicates an underuse of antimicrobials.

*** Indicates an overuse of antimicrobial.

### Efficacy of different antimicrobials in reducing CBPP related mortalities

The average case fatality rate (CFR) across all herds was estimated at 36%. Treatment with tylosin resulted in a significant reduction in mortalities (*p* = 0.035), while the reduction in mortalities following treatment with tetracycline was not significant (*p* = 0.495). Other drugs used including penicillin, and diminazen did not contribute to reduced mortality rates among treated herds (Table 2).

**Table 2:** Output from logistic regression analysis shows that tylosin significantly reduced CBPP mortality.

Drugs	Estimate	Standard error	*p*-value
Tetracycline	-0.0562	0.0793	0.4951
Tylosin	-0.2582	0.1063	0.04*
Pen & strep	0.0123	0.0961	0.9005
Diminazen	0.0505	0.0878	0.5780

## Discussion

According to the WOAH Terrestrial Animal Health Code (Chap. 11.5, CBPP) and the associated disease fact-sheet, antibiotic treatment of CBPP-infected cattle is not recommended, as it may lead to healthy looking, yet still infected and infective animals that can become long-term carriers and potential sources of infection^5^. However, despite this guidance, observation from the field indicates widespread and entrenched antimicrobial treatment among livestock keepers. In the Kenyan pastoral setting, this study observed that tetracycline and tylosin are incorrectly used through frequent under-dosing or over-dosing, without veterinary prescription or diagnosis, as an immediate remedial intervention to suspected CBPP outbreaks. This disconnect between policy and practice underscores the challenges of enforcing global standards in resource-limited scenarios, where livestock health interventions are driven more by accessibility, affordability, and perceived efficacy than by scientific or regulatory guidance. Nevertheless, judicious and evidence-based antimicrobial use remains important for managing CBPP-associated complications and other bacterial or mycoplasmal respiratory infections, when guided by proper diagnosis, dosing, and veterinary oversight^18^.

The widespread use of penicillin, streptomycin, tetracycline, and tylosin, often administered as single application or irrational sequential use reveal systemic gaps in veterinary service guidance and regulatory oversight. Notably, antimicrobials such as penicillin and diminazen were frequently used, although these drugs are not expected to be effective against *Mmm*, the CBPP causative agent, which lacks a cell wall targeted by β-lactam antibiotics^15,16^, while diminazen has a selective action on trypanosomes^19^. This pattern mirrors findings from other studies in Kenya and Ethiopia, where livestock keepers resort to empirical and often inappropriate antimicrobial therapy due to poor access to diagnostic services and limited support from veterinary extension often due to the high mobility of pastoral communities^20,21^.

In contrast to data from controlled efficacy trials^18^ and mathematical models^22^, our study found no significant improvement in the reduction of cattle mortalities following farmer-led treatment of CBPP with oxy-tetracyclines. The low efficacy of oxy-tetracycline under field conditions is attributed to a combination of potential factors such as deviation from prescribed dosage, improper storage, suboptimal-adherence to the stipulated expiry periods and potential development of bacterial resistance to oxy-tetracyclines.

The preference for antimicrobials over vaccination may be influenced by broader contextual factors, including access, affordability and awareness which can affect CBPP management in the study area. Only 34.7% of the farmers reported vaccinating their animals, with most vaccinations administered reactively during outbreaks (28/33, 84.8%) rather than preventatively. This reactive approach perpetuates endemicity, leading to recurrent outbreaks, high case fatality rates (CFR = 36%), and substantial economic losses. Such low vaccine uptake is consistent with reports from pastoral settings in Kenya, Ghana, and Ethiopia^23–25^, and are linked to misconceptions about vaccine efficacy, poor cold-chain infrastructure, cost barriers, and the logistical challenges of reaching nomadic herds. Consequently, farmers depend on antimicrobials as a more accessible and immediate solution, inadvertently increasing the AMR selection pressure within herds and the surrounding environment.

Comparable studies from Ethiopia, Niger, and South Sudan reported similar challenges in implementing sustained vaccination programs and controlling transboundary livestock movement^26,27^. In contrast, successful CBPP control in high-income countries has relied on stamping out, stringent movement controls, effective surveillance, and widespread adoption of vaccination programs that are largely absent in pastoral contexts of sub-Saharan Africa.

A major finding of this study was the high level of noncompliance (83.3%) with recommended antimicrobial withdrawal periods especially for milk-producing animals, indicating the subsequent continued consumption of milk and milk products despite the recent administration of antimicrobials. These findings align with previous work in Kenya and Tanzania, which reported high levels of tetracycline and β-lactam residues in milk samples from informal markets^28^. This noncompliance directly contributes to the presence of antimicrobial residues in milk consumed by households, thereby exposing humans to chronic low-dose antibiotic intake. Such exposure can trigger allergic reactions, toxic effects, and more importantly, selection for resistant bacterial strains in the human gut microbiota^29^. Furthermore, it may also exert a deleterious effect on beneficial bacteria, especially probiotics like bifido-bacteria and lactobacillus.

From a One Health perspective, the implications extend beyond food safety. Inappropriate antimicrobial use and environmental contamination through excreted residues contribute to the dissemination of resistance genes in soil and water systems^30,31^. The continued use of tylosin and tetracycline in livestock, for instance, promotes cross-resistance among macrolides and compounds the global human AMR challenge^32^. Thus, livestock antimicrobial use practices are intricately linked to the human health burden of AMR, particularly in resource-limited settings where regulatory enforcement and residue monitoring remain weak.

Addressing these gaps requires a multifaceted One Health strategy that bridges veterinary, public health, and environmental sectors. Strengthening veterinary governance and regulatory enforcement should be prioritized to control antimicrobial distribution and curb unprescribed use in livestock. In parallel, continuous farmer training and community sensitization on prudent antimicrobial use and biosecurity practices, coupled with increased access to laboratory diagnosis are essential to improve compliance and reduce empirical therapy. Policy emphasis should shift toward prudent use of antimicrobials and preventive vaccination as the cornerstone of CBPP control, supported by investments in cold-chain infrastructure, improved vaccine quality, and participatory delivery models that enhance trust and acceptance among pastoral communities. In parallel, public health authorities should reinforce food safety monitoring systems to detect and manage antimicrobial residues in animal-source foods such as milk and meat. Finally, there is a critical need for expanded field-based epidemiological studies to generate context-specific evidence on occurrence of major livestock respiratory diseases like CBPP, and the real-world effectiveness of current control strategies to inform policy interventions that are tailored to pastoral production systems.

## Supplementary Information

Below is the link to the electronic supplementary material.

Supplementary Material 1

## Data Availability

The datasets generated and/or analyzed during the current study are available as a supplementary material (S1_raw data_ Treatment of Contagious Bovine Pleuropneumonia as a potential driver for antimicrobial resistance in pastoral production systems of Kenya).
